# Comparisons and Impacts of the Basic Components of Sarcopenia Definition and Their Pairwise Combinations in Gastric Cancer: A Large-Scale Study in a Chinese Population

**DOI:** 10.3389/fnut.2021.709211

**Published:** 2021-10-20

**Authors:** Feng-Min Zhang, Xian-Zhong Zhang, Han-Ping Shi, Zhao Zhang, Su-Lin Wang, Zi-Le Shen, Xiao-Lei Chen, Xian Shen, Zhen Yu, Cheng-Le Zhuang

**Affiliations:** ^1^Colorectal Cancer Center and Department of Gastrointestinal Surgery, Shanghai Tenth People's Hospital, Tongji University School of Medicine, Shanghai, China; ^2^Departments of Gastrointestinal Surgery, Beijing Shijitan Hospital, Capital Medical University, Beijing, China; ^3^Department of Clinical Nutrition, Beijing Shijitan Hospital, Capital Medical University, Beijing, China; ^4^The Radiology Imaging Center, The First Affiliated Hospital, Wenzhou Medical University, Wenzhou, China; ^5^Department of Gastrointestinal Surgery, The First Affiliated Hospital, Wenzhou Medical University, Wenzhou, China; ^6^Department of Gastrointestinal Surgery, The Second Affiliated Hospital, Wenzhou Medical University, Wenzhou, China

**Keywords:** sarcopenia, muscle mass, muscle radiodensity, handgrip strength, gait speed

## Abstract

**Background and Aims:** Sarcopenia is negatively associated with clinical outcomes. However, the definitions of sarcopenia are inconsistent across international consensuses. Thus, the purpose of this study is to compare the impact of the basic definition components of sarcopenia and their combinations in post-operative complications and overall survival, aiming to find the best sarcopenia definition to stratify the prognosis in an Asian population.

**Methods:** A total of 1,307 patients who underwent curative surgery for gastric cancer from July 2014 to May 2019 were prospectively included. The basic sarcopenia components were measured pre-operatively, including low skeletal muscle mass index (LSMI), low skeletal muscle radiodensity (LSMD), low handgrip strength (LHGS), and low gait speed (LGS). Among them, LSMI and LSMD were measured using a CT post-processing software, LHGS was measured using an electronic hand dynamometer, and LGS was represented by a 6-m walk speed.

**Results:** For the single basic component, the muscle function parameters (LHGS or LGS) but not the muscle composition parameters (LSMI or LSMD) showed associations with post-operative complications and mortality. For the combination of the basic combinations, all statistically significant combinations included at least one muscle function parameter. The combination of muscle composition (LSMI or LSMD) and muscle function (LHGS or LGS) had a significantly higher area under the curve in the prediction of post-operative complications compared with the combinations of two muscle function parameters (LSMI plus LSMD) or two muscle composition parameters (LHGS plus LGS).

**Conclusions:** Compared with muscle composition parameters (LSMI and LSMD), muscle function parameters (LHGS and LGS) are better predictors of post-operative complications and overall survival, which should be considered as the principal determinant in the sarcopenia definition. The definition of sarcopenia consists of muscle function (LHGS or LGS) and muscle composition (LSMI or LSMD) separately, which is better than the combination of the two muscle function parameters (LHGS plus LGS) or two muscle composition parameters (LSMI plus LSMD).

## Introduction

Gastric cancer is the sixth most common cancer and the third leading cause of cancer-related deaths worldwide. It is often diagnosed at an advanced stage and has a low survival rate (1). Despite significant improvements in treatment in recent years, the prognosis of gastric cancer remains poor. Patients with gastric cancer often experience appetite loss, diminished food intake, and a loss of muscle mass (2, 3). Sarcopenia severely influences patients with gastric cancer and is shown to be associated with disability, reduced therapy intolerance, decreased response to cancer therapy, increased post-operative complications, poor quality of life, and a shorter duration of survival (4–7).

Sarcopenia originally referred to the loss of muscle mass but is now considered a muscle disease characterized by several features, including altered muscle composition and the decline of muscle function. However, there is an ongoing debate about the best approach to define sarcopenia due to the different combinations of basic definition components. The European Working Group on Sarcopenia in Older People (EWGSOP) put forward the first practical diagnostic criteria for sarcopenia in 2010 (8), in which sarcopenia was determined by low muscle mass accompanied by low muscle strength or low physical performance. Subsequently published guidelines have proposed similar definitions, with low muscle mass as the prerequisite (9–12).

Although a loss of muscle mass and a loss of muscle function were frequently correlated, the loss of muscle function was often more predominant than that of muscle mass (13). An increasing number of studies in recent years have shown that handgrip strength and gait speed are strong predictors of adverse clinical outcomes (14–16). Moreover, muscle quality, such as muscle radiodensity, is emerging as a new indicator for muscle composition and shows a significant association with poor clinical outcomes (17, 18). Thus, there have been heated arguments regarding the principal determinant in defining sarcopenia. In 2019, EWGSOP updated its original definition (EWGSOP2) (19), with low muscle strength replacing the role of muscle mass as the principal determinant. According to EWGSOP2, patients were considered to have probable sarcopenia when low muscle strength was detected, and the diagnosis was further confirmed by the presence of low muscle quantity or quality.

Inconsistent with EWGSOP2, the Asian Working Group for Sarcopenia 2019 (AWGS2019) retained its previous definition of sarcopenia (12) and adopted wider ranges of cut-off values for low handgrip strength and low physical performance in the updated 2019 consensus (20), which added more confusion to the clinical application of sarcopenia diagnosis due to the inconsistency between EWGSOP2 and AWGS2019. Up until now, the basic components of sarcopenia definition generally consist of two groups and four sub-groups across different consensuses, namely, muscle composition (low muscle quantity and low muscle quality) and muscle function (low muscle strength and low physical performance). However, to date, there have been no studies investigating the various impacts of different combinations of these components on post-operative outcomes and mortality in patients with gastric cancer.

The purpose of this study is to investigate the impacts of the low skeletal muscle mass index (LSMI), low skeletal muscle radiodensity (LSMD), low handgrip strength (LHGS), low gait speed (LGS), and their combinations on clinical outcomes, to determine the best sarcopenia definition to stratify the risk of post-operative complications and mortality in patients with gastric cancer.

## Materials and Methods

### Patients

Patients who underwent surgical resection with curative intent for gastric cancer at the First Affiliated Hospital of Wenzhou Medical University were prospectively enrolled in this study. The inclusion criteria were: (1) at least 18 years of age; (2) had a histologically confirmed gastric adenocarcinoma; (3) planned to receive elective curative gastric surgery; (4) had abdominal CT scans within 1 month before surgery in our hospital. The exclusion criteria were as follows: (1) had a history of cancer; (2) had a local recurrence or distant metastasis of gastric cancer; (3) was unable to undergo functional assessments due to physical or mental causes; (4) data on muscle mass and muscle quality were unavailable due to unqualified CT images. Informed consent had been signed by all participants. All the patients signed informed consent after being informed that their clinical information will be used anonymously for research. This study was approved by the ethics committees of The First Affiliated Hospital of Wenzhou Medical University and all procedures followed were in accordance with the Helsinki Declaration of 1964 and later versions.

### Assessments of Muscle Quantity and Quality

One of the gold standard methods in detecting body composition and abnormal body composition phenotypes is a CT assessment (19, 21). Both CT-derived total abdominal muscle areas and mean skeletal muscle radiodensity (SMD) were used to represent muscle quantity and quality according to EWGSOP2 and AWGS2019. The cross-sectional CT image at the third lumbar vertebra (L3) level was selected and a Hounsfield unit (HU) threshold of −29 to +150 was used to distinguish the muscle from other nearby tissues (22). To minimize measurement bias, a trained investigator (FMZ) identified the muscle, and the areas and mean SD were calculated automatically using a CT post-processing software (GE ADW 4.5). The muscle areas were divided by the square of the height to obtain the skeletal muscle mass index (SMI) (cm^2^/m^2^). Low muscle mass as represented by the LSMI was defined as <40.8 cm^2^/m^2^ for males and <34.9 cm^2^/m^2^ for females (23). Low muscle quality as represented by LSMD was defined as <38.5 HU for males and <8.6 HU for females (24).

### Assessments of Muscle Strength and Physical Performance

Handgrip strength and gait speed (GS) were used to represent muscle strength and physical performance. Handgrip strength (HGS) was measured on the dominant hand with an electronic hand dynamometer (EH101; Camry, Guangdong Province, China). The patients were seated comfortably with their shoulder adducted and neutrally rotated, their elbow flexed at 90°, and the forearm and wrist in a neutral position, and then asked to squeeze the dynamometer in their dominant hand with full force (25). According to the AWGS2019, LHGS (A-LHGS) was defined as <28 kg for males and <18 kg for females; according to the EWGSOP2, LHGS (E-LHGS) was defined as <27 kg for males and <16 kg for females.

Physical performance was assessed by the usual GS on a 6-m course (26). The patients started to walk at a normal speed under the command of an examiner. The time was recorded between the first footfall and the first foot crossing the 6-m end line. According to the AWGS2019, LGS (A-LGS) was defined as <1 m/s; according to the EWGSOP2, LGS (E-LGS) was defined as ≤ 0.8 m/s.

The HGS and GS were assessed by trained investigators (SLW and ZLS) once the patients were hospitalized, and the maximal value of the HGS and GS were recorded in three consecutive tests.

### Diagnosis of Sarcopenia

According to the AWGS2019, sarcopenia was defined as low muscle mass plus low muscle strength and/or low physical performance (20). According to the EWGSOP2, sarcopenia was defined as low muscle strength plus low muscle quantity and/or low muscle quality (19).

### Data Collection

Clinical data were collected prospectively and maintained in a digital database. For each patient, the data were collected by trained surgeons (FMZ, SLW, and ZLS), and discrepancies were solved by referring to an adjudicator (CLZ). The following data were collected: age, gender, BMI, smoking history, alcohol drinking history, reduced food intake (<50% of energy requirements >1 week, or any reduction for >2 weeks), weight loss (>5% within the past 6 months or >10% beyond 6 months), nutritional risk screening (NRS) 2002, Charlson comorbidity index (CCI) score, American Society of Anesthesiologists (ASA) score, hemoglobin concentration (anemia was defined as hemoglobin concentration <120 g/L for males and <110 g/L for females), albumin concentration (hypoalbuminemia was defined as albumin concentration <35 g/L), tumor-nodule-metastasis (TNM) stage, laparoscopic surgery, post-operative hospital stay, cost, and 30-day post-operative complications classified as Grade II or above according to the Claviene–Dindo classification (27).

### Follow-Up

All the patients received regular telephone interviews or outpatient reviews after surgery. A follow-up was conducted every 3 months for the first 2 years after surgery and once every 6 months thereafter. The content of the follow-up included post-operative life, physical examination, image logical examinations, endoscopy, and laboratory tests. Overall survival (OS) was calculated from the time of surgery to the time of death or the last follow-up. The latest follow-up date was February 2020.

### Statistical Analysis

The categorical data were represented as counts with percentages and compared using a Pearson's chi-square test or Fisher's exact test. The continuous data were represented as mean with an SD or median with an interquartile range (IQR) and compared using Student's *t*-test or Mann–Whitney *U*-test. Sarcopenia definition has four basic components, namely, LSMI, LSMD, LHGS, and LGS; however, a sarcopenia definition that consists of three and more (up to four) basic components is not practical as the incidence of sarcopenia by this definition would be very low, which is inconsistent with reality. Logistic regression analyses were used to investigate the association of the single component of sarcopenia definition (LSMI, LSMD, LHGS, and LGS) and their pairwise combinations (LSMI plus LSMD, LSMI plus LHGS, LSMI plus LGS, LSMD plus LHGS, LSMD plus LGS, and LHGS plus LGS) with 30-day post-operative complications. The Cox proportional hazard regression model was used to investigate the association of those components and their combinations with mortality. To avoid multicollinearity, the basic sarcopenia components and their combinations were included separately in the multifactor analysis model. The proportional hazards assumption was checked for all variables using Kaplan–Meier curves or Schoenfeld residual plots. To further reduce the interference of confounding factors and to verify the stability of the results, a total of three incremental models with increasing numbers of varieties were created to investigate the impact of the incremental adjustment. Model 1 was unadjusted. Model 2 was adjusted for age and gender. Model 3 was adjusted for Model 2 plus smoking history, alcohol drinking history, BMI, reduced food intake, weight loss, NRS 2002, CCI score, ASA score, anemia, hypoalbuminemia, TNM stage, and laparoscopic surgery. All analyses were performed with SPSS statistics version 23 (IBM, Armonk, NY, USA).

## Results

### Characteristics of the Patients

**Figure 1:**
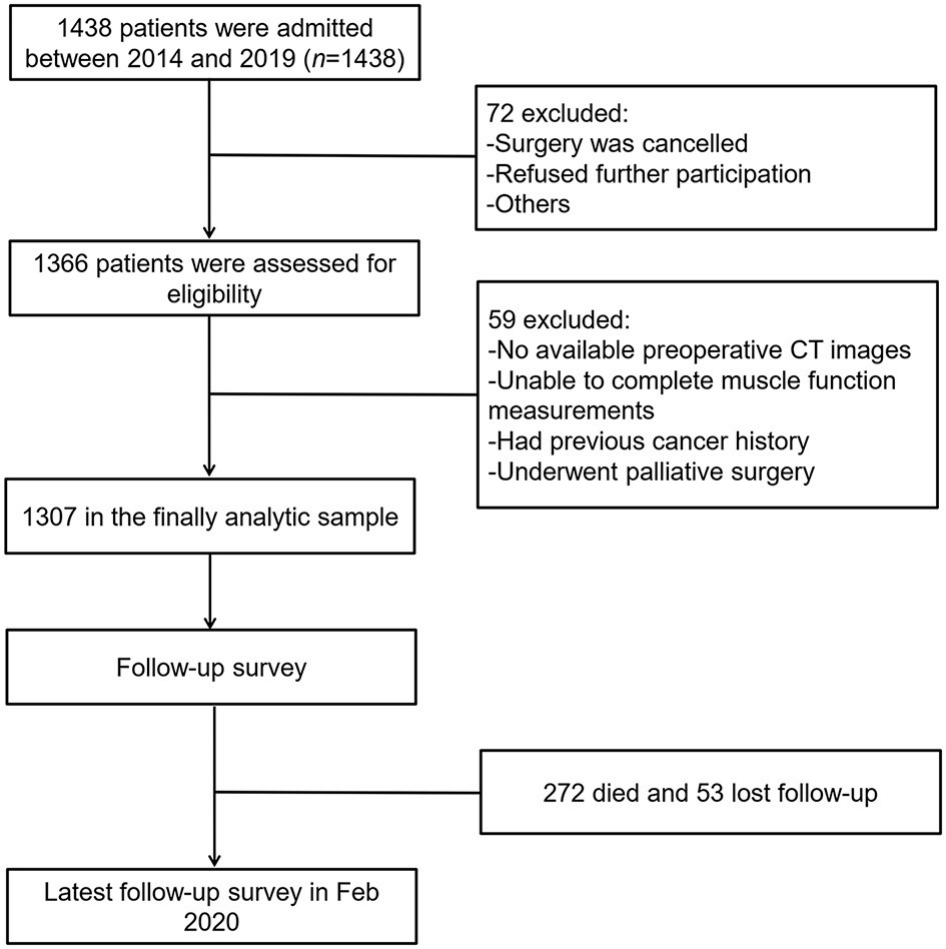
Flowchart of patient selection.

From July 2014 to May 2019, a total of 1,366 patients were enrolled in our study. Fifty-nine patients who did not meet the inclusion criteria were excluded, and 1,307 cases were analyzed. The process of patient selection is shown in Figure 1, and the different combinations of basic sarcopenia definitions are shown in Supplementary Figure 1. Of the 1,307 patients who underwent sarcopenia assessments, there were 409 with LSMI, 579 with LSMD, 480 with A-LHGS, 625 with A-LGS, 402 with E-LHGS, and 254 with E-LGS. The different combinations of the basic components of sarcopenia definition resulted in different population sizes, ranging from 127 to 334 cases (Supplementary Figure 1). Finally, 298 and 287 cases were diagnosed as AWGS2019-sarcopenia and EWGSOP2-sarcopenia, respectively.

Baseline characteristics are shown in Table 1. The median age was 66 years, and 73.6% of patients were males. Compared with the total cohort, the patients with sarcopenia tended to be older, have lower BMI, SMI, SMD, HGS, and GS, and have increased hospital stay and cost. Compared with AWGS2019-sarcopenia, the patients with EWGSOP2-sarcopenia were older, more likely to be male, and had higher BMI and SMI but lower HGS. The other clinical characteristics were similar between AWGS2019 and EWGSOP2.

**Table 1:** Patient baseline characteristics.

**Factors**	**Total**	**AWGS2019^a^-sarcopenia**	**EWGSOP2^b^-sarcopenia**	***P*-value^c^**
	**(*n* = 1,307)**	**(*n* = 298)**	**(*n* = 287)**	
Age, year	66.0 (14.0)	72.5 (11.3)	74 (10)	0.042^*^
*Gender*				0.003^*^
Male	962 (73.6)	203 (68.1)	227 (79.1)	
Female	345 (26.4)	95 (31.9)	60 (20.9)	
BMI, kg/m^2^	22.5 (4.0)	20.6 (3.5)	21.5 (4.0)	<0.001^*^
SMI, cm^2^/m^2^	42.4 (10.3)	34.7 (6.8)	38.5 (8.9)	<0.001^*^
SMD, HU	37.2 (10.1)	33.3 (9.4)	32.1 (8.6)	0.080
HGS, mean (SD), kg	27.7 (9.0)	21.8 (7.4)	19.1 (5.8)	<0.001^*^
GS, m/s	1.0 (0.3)	0.86 (0.23)	0.85 (0.29)	0.801
*Smoking*				0.402
Yes	328 (25.1)	65 (21.8)	71 (24.7)	
No	979 (74.9)	233 (78.2)	216 (75.3)	
*Alcohol drinking*				0.519
Yes	256 (19.6)	49 (16.4)	53 (18.5)	
No	1,051 (80.4)	249 (83.6)	234 (81.5)	
*Reduced food intake*				0.251
Yes	403 (30.8)	126 (42.3)	108 (37.6)	
No	904 (69.2)	172 (57.7)	179 (62.4)	
*Weight loss*				0.709
Yes	297 (22.7)	83 (27.9)	76 (26.5)	
No	1.010 (77.3)	215 (72.1)	211 (73.5)	
*NRS 2002 ≥ 3*				0.159
Yes	465 (35.6)	172 (57.7)	149 (51.9)	
No	842 (64.4)	126 (42.3)	138 (48.1)	
*CCI score*				0.472
0	960 (73.5)	212 (71.1)	191 (66.6)	
1	243 (18.5)	54 (18.1)	62 (21.6)	
≥2	104 (8.0)	32 (10.8)	34 (11.8)	
*ASA score ≥ 3*				0.933
Yes	154 (11.8)	47 (15.8)	46 (16.0)	
No	1,153 (88.2)	251 (84.2)	241 (84.0)	
*Anemia*				0.690
Yes	457 (35.0)	155 (52.0)	154 (53.7)	
No	850 (65.0)	143 (48.0)	133 (46.3)	
*Hypoalbuminemia*				0.141
Yes	305 (23.3)	114 (38.3)	127 (44.3)	
No	1,002 (76.7)	184 (61.7)	160 (55.7)	
*TNM stage*				0.731
I	491 (37.5)	87 (29.2)	76 (26.5)	
II	316 (24.2)	79 (26.5)	82 (28.6)	
III	500 (38.3)	132 (44.3)	129 (44.9)	
*Laparoscopic surgery*				0.384
Yes	470 (36.0)	95 (31.9)	82 (28.6)	
No	837 (64.0)	203 (68.1)	205 (71.4)	
Post-operative hospital stay, day	13.0 (7.0)	14.0 (9.0)	15.0 (8.0)	0.115
Cost, Yuan	59,893 (22,610)	67,266 (27,418)	68,488 (27,035)	0.248

*Numbers are median (interquartile range) or number (%), unless otherwise stated*.

* *Statistically significant (P <0.05)*.

a *Includes patients who had low SMI (LSMI) plus low HGS (LHGS) or those who had LSMI plus low GS (LGS)*.

b *Includes patients who had LHGS plus LSMI or those who had LHGS plus low SMD (LSMD)*.

c *Comparison between AWGS2019-sarcopenia and EWGSOP2-sarcopenia*.

*AWGS, Asian Working Group for Sarcopenia; EWGSOP, European Working Group on Sarcopenia in Older People; SD, standard deviation; BMI, body mass index; SMI, skeletal muscle mass index; SMD, skeletal muscle radiodensity; HU, Hounsfield unit; HGS, handgrip strength; GS, gait speed; NRS, nutritional risk screening; CCI, Charlson comorbidity index; ASA, American Society of Anesthesiologists; TNM, tumor-nodule-metastasis*.

### Impacts of the Basic Components of Sarcopenia Definition on the Post-operative Complication and Mortality

**Figure 2:**
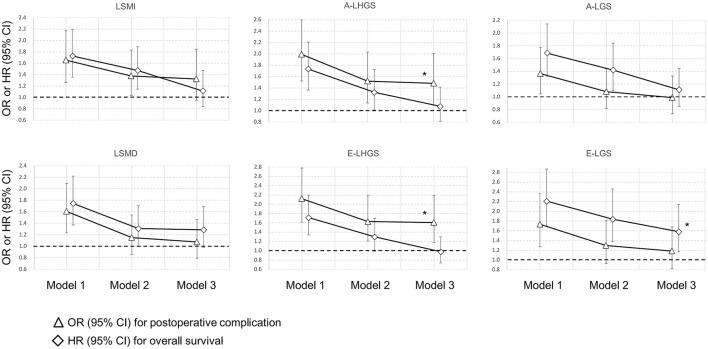
Association of basic sarcopenia components with clinical outcomes. *Statistically significant. A, Asian Working Group for Sarcopenia; E, European Working Group on Sarcopenia in Older People; LSMI, low skeletal muscle mass index; LSMD, low skeletal muscle radiodensity; LHGS, low handgrip strength; LGS, low gait speed.

The odds ratio (OR) and hazard ratio (HR) of post-operative complications and mortality for the different basic components are shown in Figure 2, with the corresponding estimates presented in Supplementary Table 1. The incidence of post-operative complications was 21.7% (284/1,307) in the total cohort. In the final model, the muscle composition parameters including LSMI and LSMD were not associated with post-operative complications nor mortality. In contrast, A-LHGS (OR = 1.481, 95% CI = 1.092–2.007, *P* = 0.011) and E-LHGS (OR = 1.606, 95% CI = 1.177–2.191, *P* = 0.003) were significantly associated with post-operative complications. The E-LGS (HR = 1.582, 95% CI = 1.169–2.142, *P* = 0.003) was significantly associated with mortality.

Considering the significant and distinct impacts of E-LHGS and E-LGS on post-operative complications and mortality, we compared the SMI and SMD between E-LHGS and E-LGS (Supplementary Table 2). We found that the patients with E-LGS had significantly lower SMI (39 vs. 40.5 cm^2^/m^2^, *P* = 0.014) and SMD (32.5 vs. 35.0 HU, *P* < 0.001) compared with E-LHGS.

### Impacts of Different Pairwise Combinations of Basic Components on the Post-operative Complication and Mortality

**Figure 3:**
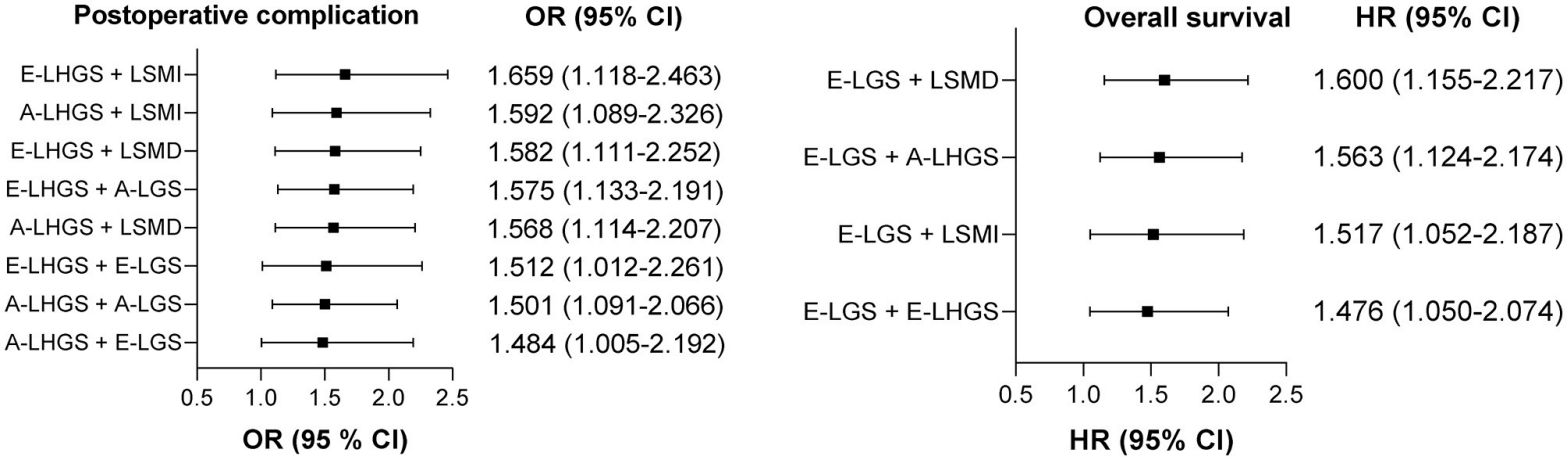
Ranking of the relationship strength of the associations between different pairwise combinations of basic components and clinical outcomes. A, Asian Working Group for Sarcopenia; E, European Working Group on Sarcopenia in Older People; LSMI, low skeletal muscle mass index; LSMD, low skeletal muscle radiodensity; LHGS, low handgrip strength; LGS, low gait speed.

The OR and HR values with statistical significance are ranked in Figure 3, with the corresponding estimates presented in Supplementary Table 3. Whether for post-operative complications or mortality, all statistically significant combinations included at least one muscle function parameter, and the strongest combinations were those that consisted of both muscle function and muscle composition. For post-operative complications, the strongest combination was E-LHGS plus LSMI (OR = 1.659, 95% CI = 1.118–2.463, *P* = 0.012). For mortality, the strongest combination was E-LGS plus LSMD (HR = 1.6, 95% CI = 1.155–2.217, *P* = 0.005). The combination of LSMI plus LSMD was neither associated with post-operative complication nor mortality.

Given the important role of muscle function, we proposed an alternative sarcopenia definition, which was defined as the presence of E-LHGS or E-LGS plus LSMI or LSMD. For post-operative complications (Table 2), the EWGSOP2-sarcopenia (OR = 1.856, 95% CI = 1.324–2.602, *P* < 0.001) and the new-definition sarcopenia (OR = 1.655, 95% CI = 1.191–2.299, *P* = 0.003) showed statistical significance in the final model. However, none of the definitions were associated with mortality in the multivariate analysis (Table 3).

**Table 2:** Impact of different sarcopenia definitions on post-operative complications.

**Factors**	**OR (95% CI)**	***P*-value**
*LSMI plus (LHGS^a^ or LGS^a^) [AWGS2019 definition]*		
Model 1	1.900 (1.420–2.544)	<0.001^*^
Model 2	1.459 (1.066–1.997)	0.018^*^
Model 3	1.416 (0.996–2.012)	0.053
*LHGS^b^ plus (LSMI or LSMD) [EWGSOP2 definition]*		
Model 1	2.595 (1.942–3.467)	<0.001^*^
Model 2	1.947 (1.413–2.683)	<0.001^*^
Model 3	1.856 (1.324–2.602)	<0.001^*^
*(LHGS^b^ or LGS^b^) plus (LSMI or LSMD)*		
Model 1	2.387 (1.811–3.147)	<0.001^*^
Model 2	1.779 (1.300–2.435)	<0.001^*^
Model 3	1.655 (1.191–2.299)	0.003^*^

* *Statistically significant (P < 0.05)*.

a *Defined by AWGS2019*.

7 *Defined by EWGSOP2*.

*Model 1 was unadjusted; Model 2 was adjusted for age and sex; Model 3 was adjusted for Model 2 plus BMI, smoking history, alcohol drinking history, reduced food intake, weight loss, NRS 2002 ≥3, CCI score, ASA score ≥3, anemia, hypoalbuminemia, TNM stage, and laparoscopic surgery*.

*AWGS, Asian Working Group for Sarcopenia; EWGSOP, European Working Group on Sarcopenia in Older People; LSMI, low skeletal muscle mass index; LSMD, low skeletal muscle radiodensity; LHGS, low handgrip strength; LGS, low gait speed; OR, odds ratio; CI, confidential interval*.

**Table 3:** Impact of different sarcopenia definitions on overall survival.

**Factors**	**HR (95% CI)**	***P*-value**
*LSMI plus (LHGS^a^ or LGS^a^) [AWGS2019 definition]*		
Model 1	2.040 (1.584–2.626)	<0.001^*^
Model 2	1.629 (1.239–2.142)	<0.001^*^
Model 3	1.236 (0.915–1.671)	0.167
*LHGS^b^ plus (LSMI or LSMD) [EWGSOP2 definition]*		
Model 1	2.084 (1.612–2.695)	<0.001^*^
Model 2	1.544 (1.159–2.058)	0.003^*^
Model 3	1.076 (0.795–1.458)	0.635
*(LHGS^b^ or LGS^b^) plus (LSMI or LSMD)*		
Model 1	1.953 (1.528–2.496)	<0.001^*^
Model 2	1.440 (1.085–1.911)	0.011^*^
Model 3	1.122 (0.835–1.508)	0.446

* *Statistically significant (P < 0.05)*.

a *Defined by AWGS2019*.

b *Defined by EWGSOP2*.

*Model 1 was unadjusted; Model 2 was adjusted for age and sex; Model 3 was adjusted for Model 2 plus BMI, smoking history, alcohol drinking history, reduced food intake, weight loss, NRS 2002 ≥3, CCI score, ASA score ≥3, anemia, hypoalbuminemia, TNM stage, and laparoscopic surgery*.

*AWGS, Asian Working Group for Sarcopenia; EWGSOP, European Working Group on Sarcopenia in Older People; LSMI, low skeletal muscle mass index; LSMD, low skeletal muscle radiodensity; LHGS, low handgrip strength; LGS, low gait speed; HR, hazard ratio; CI, confidential interval*.

**Figure 4:**
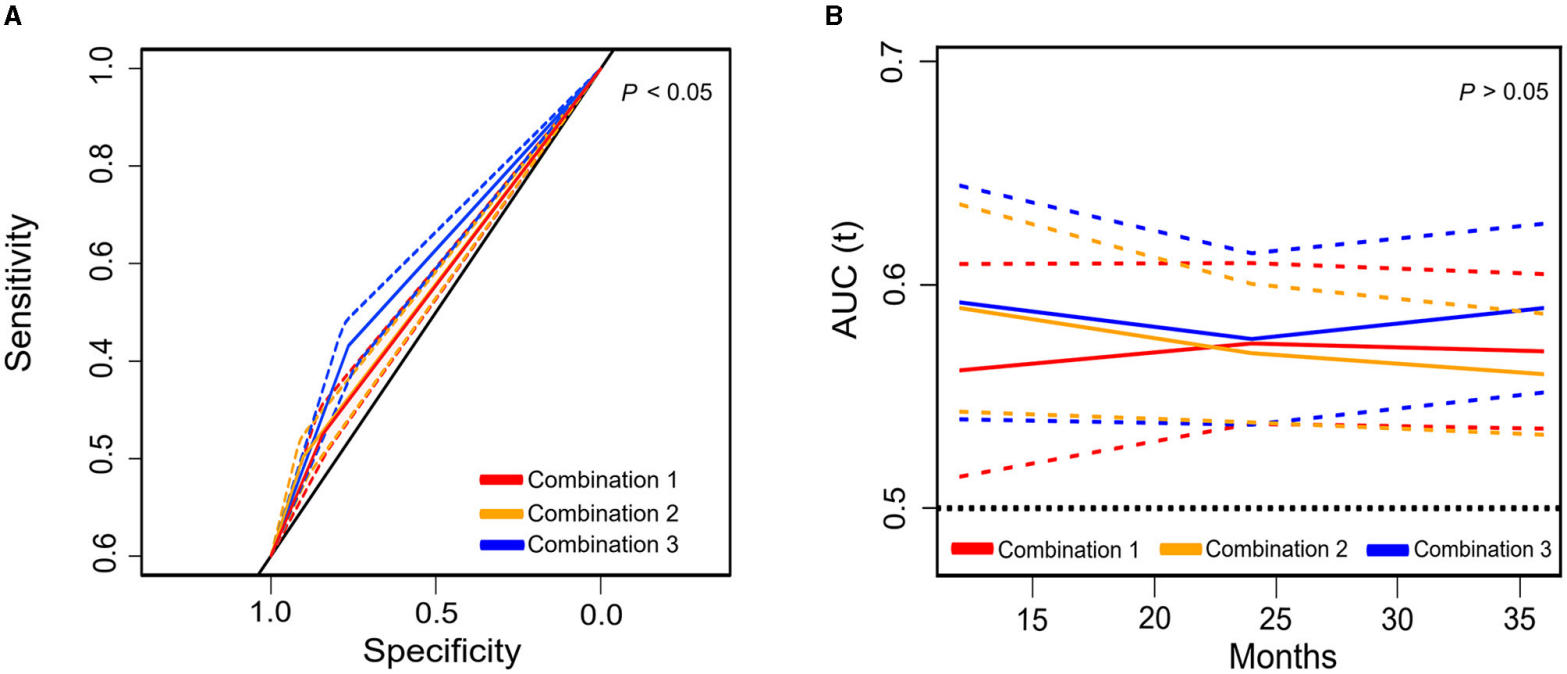
The area under the receiver operating characteristic (ROC) curve (solid line) and 95% CI (dotted line) of different pairwise combinations of basic components for post-operative complications **(A)** and mortality **(B)**. Combination 1: LSMD plus LSMI; Combination 2: E-LHGS plus E-LGS; Combination 3: (E-LHGS or E-LGS) plus (LSMD or LSMI); E, European Working Group on Sarcopenia in Older People; ROC, receiver operating characteristic curve; LSMI, low skeletal muscle mass index; LSMD, low skeletal muscle radiodensity; LHGS, low handgrip strength; LGS, low gait speed; CI, confidential interval.

Additionally, to examine the impacts of these pairwise combinations on predicting post-operative complications and mortality, a receiver operating characteristic (ROC) curve was performed (Figure 4) with the corresponding estimates presented in Supplementary Tables 4, 5. The combination of muscle function (LSMI or LSMD) plus muscle composition (LHGS or LGS) had a significantly higher area under the curve [0.598, 95% CI = 0.57–0.626)] in the prediction of post-operative complications compared with the combinations of the two muscle function parameters (LSMI plus LSMD) [0.546, 95% CI = 0.517–0.575)] or two muscle composition parameters (LHGS plus LGS) [0.553, 95% CI = 0.524–0.581)]. These three types of combinations had no statistical difference in predicting mortality, but the combination of muscle composition plus muscle function showed a trend of a higher area under the ROC curve (AUC) in a longer follow-up period.

## Discussion

In the current study, we demonstrated that the muscle function measured as LHGS and LGS was a better predictor of post-operative complications and mortality than the muscle composition measured as LSMI and LSMD. Additionally, we found that LHGS is a strong predictor of post-operative complications, while LGS is a strong predictor of mortality. Moreover, the sarcopenia definition consisting of both muscle function parameters (LHGS or LGS) and muscle composition parameters (LSMI or LSMD) had stronger impacts on the clinical outcomes when compared to the combinations of two muscle function parameters (LHGS plus LGS) or two muscle composition parameters (LSMI plus LSMD). These findings suggest that muscle function should be considered as the principal determinant in the diagnosis of sarcopenia, and muscle composition is also necessary for the diagnosis.

Previous studies have examined the association of muscle function and muscle composition with clinical outcomes. Sato et al. showed that pre-operative LHGS but not low lean body mass was significantly associated with grade 2 or higher morbidities in patients who underwent curative surgery for gastric cancer (28). A large, multiethnic, national study showed that LGS defined by <0.8m/s had a stronger association with death in the elderly compared with low lean mass (29). In our study, we obtained similar results, in which muscle function was found to be a better indicator as compared with muscle composition to predict post-operative complications and mortality. Notably, LHGS and LGS had different impacts on clinical outcomes; LHGS was strongly associated with post-operative complications, while LGS was strongly associated with mortality.

With respect to the different impacts of LHGS and LGS on clinical outcomes, Revenig et al. found that LHGS but not LGS showed a significant association with short-term morbidity and mortality in patients who underwent major abdominal operations (30). On the other hand, the HUNT II study reported no association of the different tertiles of HGS with mortality (31). An observational study conducted on more than 500,000 participants found that slow walking speed but not weak handgrip strength was associated with an increased risk of mortality in the low-BMI subset (15). A recent study also reported that it was LGS but not LHGS that showed significant association with mortality (32).

Although the exact reason for the different impacts of LHGS and LGS is unclear, this phenomenon may be partly explained in two ways. First, HGS as a form of explosive isometric force is significantly associated with the capacity of substrate reservation and utilization (33), systemic inflammation (34), and abnormal metabolism (35). Patients with LHGS are more likely to have increased complications due to their poor adaptability to surgical strikes. Second, LGS accounts for the 70% increase in the disability of cancer patients (32). The loss of mobility is likely to lead to the vicious cycle of decreased physical activity and contributes directly to a higher risk of mortality (26). Our analysis revealed that patients with LGS had worse muscle conditions than those with LHGS, which justified the hypothesis that the patients with LGS had worse survival due to their poor physical fitness. In the EWGSOP2, gait speed only serves as a severity indicator of sarcopenia diagnosis. In our study, LGS defined by the EWGSOP2 (<0.8 m/s), but not AWGS2019 (<1 m/s), and was found to be an independent risk factor for mortality. Our study indicated that LGS alone and its combination with muscle composition (LSMI or LSMD) were strong predictors of adverse clinical outcomes, which should also be considered as the prerequisite of sarcopenia definition like LHGS.

Few studies explored different combinations of the basic components of sarcopenia definition. Rodrigues et al. found that LSMI plus LSMD had the strongest association with 1-year mortality compared with LSMI alone or LSMD alone (36). However, their study did not adjust the nutrition-related variables in the analysis model, such as reduced food intake, weight loss, NRS 2002, anemia, and hypoalbuminemia. In our study, LSMI plus LSMD showed a higher risk of mortality than LSMI alone or LSMD alone in the unadjusted model, and the model adjusted for sex and age. However, the association attenuated to insignificance in the final model, which included more nutritional parameters.

Although LSMI or LSMD alone were not related to clinical outcomes, we found that their combinations with LHGS and LGS improved the predictive effect of LHGS alone or LGS alone in post-operative complications and mortality. In concordance with our study, Gan et al. found that the risk of non-alcoholic fatty liver disease increased when low muscle mass and LHGS were simultaneously detected compared with only one of them detected (37). Furthermore, Maurício et al. demonstrated that low muscle mass in combination with low muscle strength instead of other nutritional parameters had the strongest association with complications in patients with colorectal cancer (38).

However, the existing evidence is scattered. There is a paucity of previous studies to systematically compare the impacts of different combinations of the basic components of sarcopenia definition on clinical outcomes in one cohort. No study has yet discussed the impacts of LSMD plus LHGS, LSMD plus LGS, and LHGS plus LGS on clinical outcomes. Our findings extended the previous evidence by reporting that muscle function should be considered as the principal determinant in the diagnosis of sarcopenia. Sarcopenia defined by both muscle function and muscle composition had a stronger impact on the clinical outcomes when compared with that of muscle function alone or muscle composition alone. Our results indicated that the combination of muscle function plus muscle composition had the best ability to predict post-operative complications and a trend of higher mortality prediction ability in a longer follow-up period when compared with the combination of muscle composition plus muscle composition or muscle function plus muscle function.

Our results are supported by interventional research. With the development of perioperative management, pre-operative functional intervention gained increasing attention in the team-based approach and the enhanced recovery after surgery pathway. Pre-habilitation combining endurance and resistance training has been shown to improve physical capacity and muscle strength and decrease post-operative complications (39–41). Patients who do more exercise were observed to have a lower risk of mortality and recurrence (42). The associations of LHGS and LGS with clinical outcomes reported in our study emphasize the importance of pre-habilitation and rehabilitation in patients with gastric cancer.

The present study has some potential limitations. First, the observational design of our study does not allow us to draw firm conclusions on the causal role of muscle function and muscle composition in post-operative complications or mortality. However, data were prospectively collected to minimize the recall bias and many potential confounding factors were adjusted, including BMI, reduced food intake, and weight loss, which are strong predictors of clinical outcomes in patients who underwent abdominal operations (43). Second, the cut-off values for LSMI and LSMD were obtained from our previous large-scale studies (23, 24) due to the lack of a unified standard on the cut-off values for CT-assessed LSMI and LSMD. The existing cut-off values were mainly derived from populations that were not Chinese (44, 45). We believe that using cut-off values from Chinese-specific large sample studies can yield more accurate results under the consideration of the race differences between Chinese and other populations. Third, the analysis of this study was conducted in patients with gastric cancer, which may limit the generalization of the conclusion. Fourth, we were unable to calculate the sensitivity and specificity of our definitions in the present study because sarcopenia currently lacks a gold standard.

In conclusion, this study found that muscle function has stronger impacts on clinical outcomes compared with muscle composition. Low handgrip strength is a strong predictor of post-operative complications, and LGS is a strong predictor of mortality. The sarcopenia definition that consists of both muscle function and muscle composition showed the strongest impacts on clinical outcomes. These findings suggested that the definition of sarcopenia should be constructed using muscle function as the principal determinant and that this should be used together with muscle composition.

## Data Availability Statement

The raw data supporting the conclusions of this article will be made available by the authors, without undue reservation.

## Ethics Statement

The studies involving human participants were reviewed and approved by Ethics Committee for Clinical Research of the First Affiliated Hospital of Wenzhou Medical University. The patients/participants provided their written informed consent to participate in this study.

## Author Contributions

C-LZ and ZY designed the study. ZZ provided technical support. F-MZ, S-LW, and Z-LS collected the data. X-ZZ, ZY, X-LC, and XS did the analysis and interpretation of data. F-MZ and H-PS wrote the article. X-ZZ and ZY revised the article and took the decision to submit the article for publication. All authors contributed to the article and approved the submitted version.

## Funding

This work was funded by the National Natural Science Foundation of China (Nos. 81800795 and 81770884), the Shanghai Municipal Commission of Health and Family Planning (No. 20184Y0301), and the Key Technology of Palliative Care and Nursing for Cancer Patients (2017YFC1309200).

## Conflict of Interest

The authors declare that the research was conducted in the absence of any commercial or financial relationships that could be construed as a potential conflict of interest.

## Publisher's Note

All claims expressed in this article are solely those of the authors and do not necessarily represent those of their affiliated organizations, or those of the publisher, the editors and the reviewers. Any product that may be evaluated in this article, or claim that may be made by its manufacturer, is not guaranteed or endorsed by the publisher.
