# Supplementary material for: Comparisons and Impacts of the Basic Components of Sarcopenia Definition and Their Pairwise Combinations in Gastric Cancer: A Large-Scale Study in a Chinese Population
Source: Front Nutr. 2021 Oct 20;8:709211. doi: 10.3389/fnut.2021.709211 (PMC8564036; doi:10.3389/fnut.2021.709211)
Supplement: Supplementary file 1 [file Data_Sheet_1.docx]

Supplementary Figure 1 Combinations of basic components of sarcopenia definition. AWGS2019 (A), Asian Working Group for Sarcopenia; EWGSOP2 (E), European Working Group on Sarcopenia in Older People; LSMI, low skeletal muscle mass index; LSMD, low skeletal muscle radiodensity; LHGS, low handgrip strength; LGS, low gait speed.


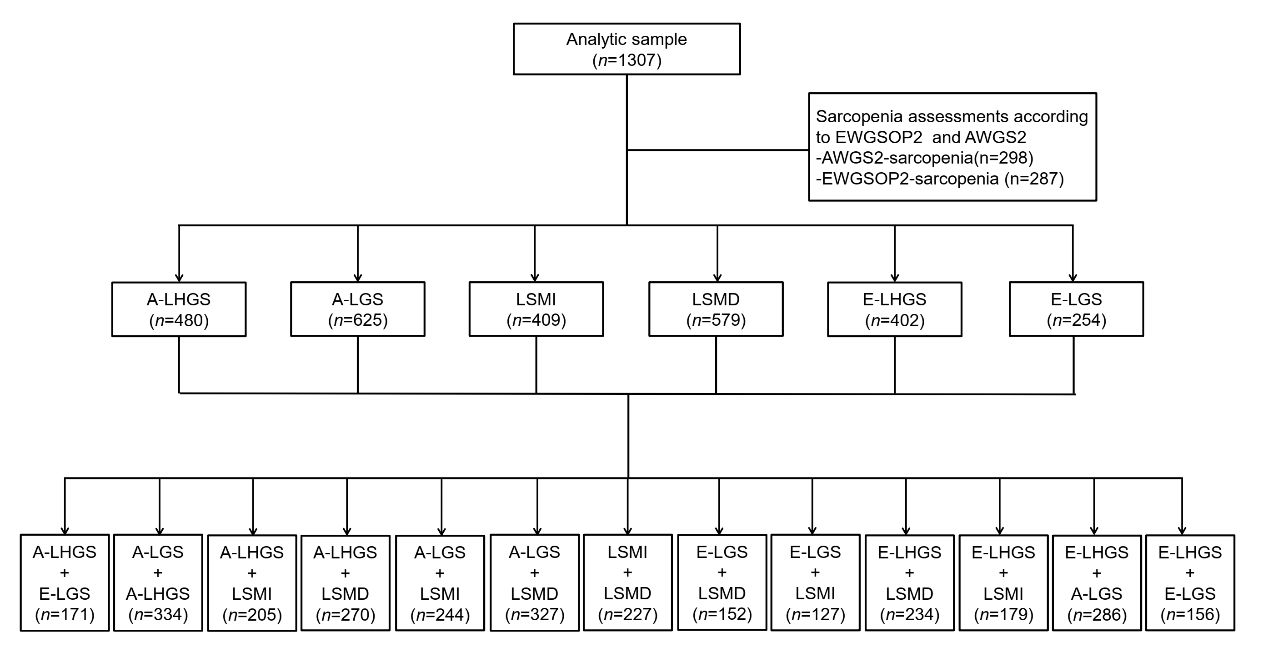


| Supplementary Table 1 Impact of basic sarcopenia components on postoperative complication and overall survival according to AWGS2019 and EWGSOP2 | | | | |
| --- | --- | --- | --- | --- |
| Factors | Postoperative complication | | Overall survival | |
|  | OR (95% CI) | *P* value | HR (95% CI) | *P* value |
| LSMI |  |  |  |  |
| Model 1 | 1.655 (1.259-2.175) | < 0.001^*^ | 1.728 (1.357-2.199) | < 0.001^*^ |
| Model 2 | 1.377 (1.035-1.832) | 0.028^*^ | 1.474 (1.148-1.893) | 0.002^*^ |
| Model 3 | 1.326 (0.953-1.846) | 0.094 | 1.113 (0.840-1.475) | 0.455 |
| LSMD |  |  |  |  |
| Model 1 | 1.607 (1.234-2.093) | < 0.001^*^ | 1.745 (1.371-2.221) | < 0.001^*^ |
| Model 2 | 1.150 (0.857-1.544) | 0.352 | 1.308 (1.000-1.710) | 0.050 |
| Model 3 | 1.076 (0.792-1.463) | 0.638 | 1.286 (0.979-1.689) | 0.071 |
| A-LHGS |  |  |  |  |
| Model 1 | 1.989 (1.524-2.597) | < 0.001^*^ | 1.733 (1.361-2.208) | < 0.001^*^ |
| Model 2 | 1.519 (1.136-2.032) | 0.005^*^ | 1.321 (1.014-1.720) | 0.039^*^ |
| Model 3 | 1.481 (1.092-2.007) | 0.011^*^ | 1.074 (0.815-1.415) | 0.612 |
| A-LGS |  |  |  |  |
| Model 1 | 1.363 (1.047-1.775) | 0.021^*^ | 1.684 (1.323-2.143) | < 0.001^*^ |
| Model 2 | 1.082 (0.814-1.438) | 0.588 | 1.420 (1.097-1.838) | 0.008^*^ |
| Model 3 | 0.988 (0.736-1.326) | 0.934 | 1.110 (0.851-1.448) | 0.441 |
| E-LHGS |  |  |  |  |
| Model 1 | 2.117 (1.613-2.779) | < 0.001^*^ | 1.713 (1.336-2.195) | < 0.001^*^ |
| Model 2 | 1.625 (1.211-2.182) | 0.001^*^ | 1.296 (0.989-1.698) | 0.060 |
| Model 3 | 1.606 (1.177-2.191) | 0.003^*^ | 0.979 (0.736-1.301) | 0.881 |
| E-LGS |  |  |  |  |
| Model 1 | 1.735 (1.274-2.361) | < 0.001^*^ | 2.209 (1.701-2.869) | < 0.001^*^ |
| Model 2 | 1.297 (0.926-1.818) | 0.130 | 1.839 (1.375-2.460) | < 0.001^*^ |
| Model 3 | 1.182 (0.831-1.681) | 0.351 | 1.582 (1.169-2.142) | 0.003^*^ |
| ^*^ Statistically significant (*P* < 0.05) | | | | |
| Model 1 was unadjusted; Model 2 was adjusted for age and sex; Model 3 was adjusted for Model 2 plus BMI, smoking history, alcohol drinking history, reduced food intake, weight loss, NRS 2002 ≥ 3, CCI score, ASA score ≥ 3, anemia, hypoalbuminemia, TNM stage, and laparoscopic surgery | | | | |
| AWGS (A), Asian Working Group for Sarcopenia; EWGSOP (E), European Working Group on Sarcopenia in Older People; LSMI, low skeletal muscle mass index; LSMD, low skeletal muscle radiodensity; LHGS, low handgrip strength; LGS, low gait speed; OR, odds ratio; HR, hazard ratio; CI, confidential interval | | | | |

| Supplementary Table 2 Muscle characteristics between LHGS^a^ and LGS^a^ | | | |
| --- | --- | --- | --- |
| Factors | LHGS^a^  (n=402) | LGS^a^  (n=254) | *P* value |
| SMI, cm^2^/m^2^ | 40.5 (10.2) | 39.0 (9.2) | 0.014^*^ |
| SMD, HU | 35.0 (10.1) | 32.5 (10.5) | < 0.001^*^ |
| Numbers are median (interquartile range) | | | |
| ^a^ Defined by EWGSOP2 | | | |
| ^*^ Statistically significant (P < 0.05) | | | |
| LHGS, low handgrip strength; LGS, low gait speed; SMI, skeletal muscle mass index; SMD, skeletal muscle radiodensity; HU, hounsfield unit | | | |

| Supplementary Table 3 Impact of different combinations of two basic sarcopenia components on postoperative complication and overall survival according to AWGS2019 and EWGSOP2 | | | | |
| --- | --- | --- | --- | --- |
| Factors | Postoperative complication | | Overall survival | |
|  | OR (95% CI) | *P* value | HR (95% CI) | *P* value |
| A-LHGS+E-LGS |  |  |  |  |
| Model 1 | 2.201 (1.556-3.112) | < 0.001^*^ | 2.629 (1.972-3.506) | < 0.001^*^ |
| Model 2 | 1.624 (1.119-2.357) | 0.011^*^ | 2.131 (1.577-2.915) | < 0.001^*^ |
| Model 3 | 1.484 (1.005-2.192) | 0.047^*^ | 1.563 (1.124-2.174) | 0.008^*^ |
| A-LHGS+LSMI |  |  |  |  |
| Model 1 | 2.211 (1.599-3.058) | < 0.001^*^ | 2.113 (1.598-2.795) | < 0.001^*^ |
| Model 2 | 1.645 (1.165-2.324) | 0.005^*^ | 1.616 (1.200-2.177) | 0.002^*^ |
| Model 3 | 1.592 (1.089-2.326) | 0.016^*^ | 1.175 (0.845-1.634) | 0.337 |
| A-LHGS+LSMD |  |  |  |  |
| Model 1 | 2.381 (1.771-3.200) | < 0.001^*^ | 2.099 (1.621-2.718) | < 0.001^*^ |
| Model 2 | 1.710 (1.228-2.381) | 0.002^*^ | 1.529 (1.142-2.047) | 0.004^*^ |
| Model 3 | 1.568 (1.114-2.207) | 0.010^*^ | 1.153 (0.854-1.556) | 0.353 |
| A-LGS+LSMI |  |  |  |  |
| Model 1 | 1.955 (1.435-2.663) | < 0.001^*^ | 2.133 (1.641-2.774) | < 0.001^*^ |
| Model 2 | 1.502 (1.078-2.092) | 0.016^*^ | 1.712 (1.287-2.277) | < 0.001^*^ |
| Model 3 | 1.411 (0.981-2.029) | 0.063 | 1.250 (0.920-1.699) | 0.154 |
| A-LGS+LSMD |  |  |  |  |
| Model 1 | 1.946 (1.465-2.586) | < 0.001^*^ | 2.050 (1.605-2.619) | < 0.001^*^ |
| Model 2 | 1.392 (1.017-1.905) | 0.039^*^ | 1.547 (1.177-2.035) | 0.002^*^ |
| Model 3 | 1.209 (0.872-1.678) | 0.255 | 1.260 (0.951-1.669) | 0.107 |
| A-LHGS+A-LGS |  |  |  |  |
| Model 1 | 2.076 (1.566-2.753) | < 0.001^*^ | 1.809 (1.401-2.335) | < 0.001^*^ |
| Model 2 | 1.595 (1.174-2.167) | 0.003^*^ | 1.398 (1.060-1.845) | 0.018^*^ |
| Model 3 | 1.501 (1.091-2.066) | 0.013^*^ | 1.045 (0.782-1.395) | 0.767 |
| LSMI+LSMD |  |  |  |  |
| Model 1 | 1.665 (1.207-2.297) | 0.002^*^ | 2.168 (1.670-2.816) | < 0.001^*^ |
| Model 2 | 1.172 (0.830-1.656) | 0.368 | 1.655 (1.247-2.198) | < 0.001^*^ |
| Model 3 | 1.046 (0.724-1.512) | 0.809 | 1.278 (0.953-1.713) | 0.101 |
| E-LHGS+E-LGS |  |  |  |  |
| Model 1 | 2.267 (1.585-3.242) | < 0.001^*^ | 2.621 (1.948-3.528) | < 0.001^*^ |
| Model 2 | 1.658 (1.131-2.430) | 0.010^*^ | 2.099 (1.521-2.896) | < 0.001^*^ |
| Model 3 | 1.512 (1.012-2.261) | 0.044^*^ | 1.476 (1.050-2.074) | 0.025^*^ |
| E-LHGS+LSMI |  |  |  |  |
| Model 1 | 2.297 (1.636-3.225) | < 0.001^*^ | 2.237 (1.675-2.989) | < 0.001^*^ |
| Model 2 | 1.684 (1.173-2.417) | 0.005^*^ | 1.690 (1.240-2.303) | < 0.001^*^ |
| Model 3 | 1.659 (1.118-2.463) | 0.012^*^ | 1.142 (0.812-1.606) | 0.446 |
| E-LHGS+LSMD |  |  |  |  |
| Model 1 | 2.385 (1.751-3.247) | < 0.001^*^ | 2.026 (1.546-2.655) | < 0.001^*^ |
| Model 2 | 1.692 (1.202-2.384) | 0.003^*^ | 1.440 (1.064-1.950) | 0.018^*^ |
| Model 3 | 1.582 (1.111-2.252) | 0.011^*^ | 1.058 (0.775-1.442) | 0.723 |
| E-LGS+LSMI |  |  |  |  |
| Model 1 | 2.161 (1.462-3.192) | < 0.001^*^ | 2.540 (1.858-3.472) | < 0.001^*^ |
| Model 2 | 1.567 (1.032-2.380) | 0.035^*^ | 2.054 (1.462-2.885) | < 0.001^*^ |
| Model 3 | 1.404 (0.899-2.194) | 0.136 | 1.517 (1.052-2.187) | 0.026^*^ |
| E-LGS+LSMD |  |  |  |  |
| Model 1 | 2.293 (1.598-3.290) | < 0.001^*^ | 2.630 (1.980-3.493) | < 0.001^*^ |
| Model 2 | 1.577 (1.067-2.331) | 0.022^*^ | 1.987 (1.451-2.721) | < 0.001^*^ |
| Model 3 | 1.389 (0.924-2.086) | 0.114 | 1.600 (1.155-2.217) | 0.005^*^ |
| E-LHGS+A-LGS |  |  |  |  |
| Model 1 | 2.190 (1.634-2.933) | < 0.001^*^ | 1.826 (1.400-2.380) | < 0.001^*^ |
| Model 2 | 1.668 (1.217-2.285) | 0.001^*^ | 1.393 (1.046-1.856) | 0.023^*^ |
| Model 3 | 1.575 (1.133-2.191) | 0.007^*^ | 0.961 (0.712-1.297) | 0.793 |
| ^*^ Statistically significant (P < 0.05) | | | | |
| Model 1 was unadjusted; Model 2 was adjusted for age and sex; Model 3 was adjusted for Model 2 plus BMI, smoking history, alcohol drinking history, reduced food intake, weight loss, NRS 2002 ≥ 3, CCI score, ASA score ≥ 3, anemia, hypoalbuminemia, TNM stage, and laparoscopic surgery | | | | |
| AWGS (A), Asian Working Group for Sarcopenia; EWGSOP (E), European Working Group on Sarcopenia in Older People; LSMI, low skeletal muscle mass index; LSMD, low skeletal muscle radiodensity; LHGS, low handgrip strength; LGS, low gait speed; OR, odds ratio; HR, hazard ratio; CI, confidential interval | | | | |

| Supplementary Table 4 Area under the ROC curve and 95% CI of different combinations of sarcopenia components for postoperative complication | | | | |
| --- | --- | --- | --- | --- |
| Factors | Value | 95% CI | Post-hoc pairwise comparison | *P* values |
| LSMI plus LSMD | 0.546 | 0.517-0.575 | - | - |
|  | - | - | LHGS^a^ plus LGS^a^ | 0.705 |
|  | - | - | (LHGS^a^ or LGS^a^) plus (LSMI or LSMD) | 0.003^*^ |
| LHGS^a^ plus LGS^a^ | 0.553 | 0.524-0.581 | - | - |
|  | - | - | LSMI plus LSMD | 0.705 |
|  | - | - | (LHGS^a^ or LGS^a^) plus (LSMI or LSMD) | 0.004^*^ |
| (LHGS^a^ or LGS^a^) plus (LSMI or LSMD) | 0.598 | 0.570-0.626 | - | - |
|  | - | - | LSMI plus LSMD | 0.003^*^ |
|  | - | - | LHGS^a^ plus LGS^a^ | 0.004^*^ |
| ^a^ Defined by EWGSOP2 | | | | |
| ^*^ Statistically significant (P < 0.05) | | | | |
| ROC, receiver operating characteristic curve; LSMI, low skeletal muscle mass index; LSMD, low skeletal muscle radiodensity; LHGS, low handgrip strength; LGS, low gait speed; CI, confidential interval | | | | |

| Supplementary Table 5 Area under the ROC curve and 95% CI of different combinations of sarcopenia components for overall survival | | | | | | |
| --- | --- | --- | --- | --- | --- | --- |
| Follow-up  Factors | At 12 months | | At 24 months | | At 36 months | |
|  | Value | 95% CI | Value | 95% CI | Value | 95% CI |
| LSMI plusLSMD | 0.562 | 0.506-0.618 | 0.574 | 0.532-0.616 | 0.570 | 0.530-0.611 |
| LHGS^a^ plus LGS^a^ | 0.590 | 0.543-0.636 | 0.570 | 0.538-0.600 | 0.560 | 0.532-0.587 |
| (LHGS^a^ or LGS^a^) plus (LSMI or LSMD) | 0.592 | 0.540-0.644 | 0.576 | 0.537-0.614 | 0.590 | 0.552-0.627 |
| ^a^ Defined by EWGSOP2 | | | | | | |
| All pairwise comparison did not reach statistical significance | | | | | | |
| ROC, receiver operating characteristic curve; LSMI, low skeletal muscle mass index; LSMD, low skeletal muscle radiodensity; LHGS, low handgrip strength; LGS, low gait speed; CI, confidential interval | | | | | | |
